# Effect of intravenous lidocaine on the ED_50_ of propofol for inserting gastroscope without body movement in adult patients: a randomized, controlled study

**DOI:** 10.1186/s12871-022-01861-9

**Published:** 2022-10-17

**Authors:** Xiu-Ru Qi, Jing-Yi Sun, Li-Xin An, Ke Zhang

**Affiliations:** Department of Anesthesia, Beijing Friendship Hospital, Capital Medical University, No.95 Yongan Road, Xicheng District, Beijing, 100050 China

**Keywords:** ED_50_, Lidocaine, Propofol, Gastroscope

## Abstract

**Background:**

Circulatory and respiratory depression are common  problems that occur in propofol alone sedation during gastroscopy. As a widely used analgesic adjuvant, intravenous lidocaine can reduce the consumption of propofol during Endoscopic retrograde cholangiopancreatography (ERCP) or colonoscopy. However, it is still unknown the median effective dose (ED_50_) of propofol when combined with lidocaine intravenously. This study aimed to compare the ED_50_ of propofol with or without intravenous lidocaine for inserting gastrointestinal endoscope successfully.

**Methods:**

Fifty nine patients undergoing gastroscopy or gastrointestinal (GI) endoscopy were randomly divided into control group (Group C, normal saline + propofol) or lidocaine group (Group L, lidocaine + propofol). Patients were initially injected a bolus of 1.5 mg/kg lidocaine in Group L, whereas equivalent volume of 0.9% saline in Group C. Anaesthesia was then induced with a single bolus of propofol in all subjects. The induction dose of propofol was determined by the modified Dixon’s up-and-down method, and the initial dose was 1.5 mg/kg in both groups. The primary outcome was the ED_50_ of propofol induction dose with or without intravenous lidocaine. The secondary outcomes were the induction time, the first propofol bolus time (FPBT: from MOAA/S score ≤ 1 to first rescue bolus propofol), and adverse events (AEs: hypoxemia, bradycardia, hypotension, and body movements).

**Results:**

Totally, 59 patients were enrolled and completed this study. The ED_50_ of propofol combined with lidocaine was 1.68 ± 0.11 mg/kg, significantly reduced compared with the normal saline group, 1.88 ± 0.13 mg/kg (*P* = 0.002). There was no statistical difference in induction time (*P* = 0.115) and the FPBT (*P* = 0.655) between the two groups. There was no significantly difference about the AEs between the two groups.

**Conclusion:**

The ED_50_ of propofol combined with intravenous lidocaine for successful endoscope insertion in adult patients, was 1.68 ± 0.11 mg/kg significantly reduced compared with the control group.

**Trial registration:**

Chinese Clinical Trial Registry, No: ChiCTR2200059450. Registered on 29 April 2022. Prospective registration. http://www.chictr.org.cn.

## Background

Gastroscopy has been the most common and invaluable method to diagnose and treat upper gastrointestinal (GI) diseases. Actually, patients often feel throat pain, nausea, bloating, visceral pain during biopsy and fear during the procedure without giving any sedative, leading to failure or stop-page [1]. The use of sedative and analgesic agents results in more comfortable experience, higher lesion detection rate, higher patient and endoscopist satisfaction [2]. In recent years, 98% patients in the United States choose painless gastroscopy [3]. Although different in Europe [4] and China [1], the comfortable medical treatment of endoscopy has become a general trend.

Propofol has become the most popular sedative agent because of its short onset time and quick recovery for outpatient gastroscopy [2]. However, due to the narrow therapeutic window and absence of effective antagonist, when administered alone for painless endoscopy, propofol may lead to some serious problems such as: circulatory depression and hypoxemia on the one hand [5], or gastroscope insertion failure, cough, reflux and even laryngeal spasm on the other hand [6, 7].

In order to reduce the serious problems, adjuvant drugs such as opioids, midazolam, dexmedetomidine, ketamine and so on combined with propofol are always used in endoscopy sedation. However, the combination with opioids results in better analgesia, less cough, and lower propofol dosage, more severe respiratory depression [8–10]; the combination with ketamine significantly reduces the respiratory and circulatory depression, schizophrenia-like symptoms often occurs [11]; otherwise, the combination with dexmedetomidine improves sedative and analgesic effect for its anti-anxiety and sedative properties, and less respiratory side effects, even causes prolonged hypo-tension or bradycardia in low dose [12, 13]. Obviously, these methods of medication have their own drawbacks.

Interestingly, lidocaine is not only a short-acting local anesthetics, but also has analgesic and sedative effects when injected intravenously [14]. Owing to its auxiliary analgesia effect, the total demand of propofol for painless colonoscopy was reduced [15]. It may also suppress bronchial hypersensitivity reactions induced by mechanical and thermal stimuli, or irritants (particles, gases, and blood, etc.). In addition, it may abolish the cough reflex caused by fentanyl and reduce propofol injection pain [16]. Therefore, it has been increasingly used as an adjuvant drug in sedation and anesthesia, especially for painless gastrointestinal endoscopy.

Although Liu [17] et al. already found that the ED_50_ of propofol combined with intravenous dropped from 2.01 mg/kg to 1.69 mg/kg, sufentanil also worked. So, we aim to estimate the ED_50_ of propofol combined with intravenous lidocaine, for inserting gastrointestinal endoscope successfully.

## Methods

### Ethics statement

This prospective, double-blinded randomized controlled trial was conducted in Beijing Friendship Hospital of Capital Medical University. This study protocol was approved by the Ethics Committee of Beijing Friendship Hospital, Capital Medical University (No. 2022-P2–026-02, 6 April 2022) and was registered with in the Chinese Clinical Trial Registry (http://www.chictr.org.cn; registration number: ChiCTR2200059450, 29 April 2022). This trial adhered to the applicable CONSORT guidelines.

### Inclusion and exclusion criteria

Patients scheduled for painless gastroscopy were recruited and signed the informed consent if they meet the following inclusion criteria: 18–50 years old; American Society of Anesthesiologists (ASA) Physical Statuses Grades I–II; 18 kg/m^2^ ≤ BMI ≤ 30 kg/m^2^; STOP-Bang score ≤ 5; heart rate > 50 beats/min without the history of atrioventricular block; 90 mmHg ≤ systolic blood pressure ≤ 170 mmHg; diastolic blood pressure ≤ 100 mmHg; liver and kidney function well; without local anesthetic in the past 24 hours; without analgesics and hypnotics in the past 7 days; undergoing painless gastroscopy; sign the informed consent. All participants in this study were required to sign the informed consent form.

Exclusion criteria were as follows: ① participation in other clinical trials within the past four weeks; ② allergic to lidocaine; ③ pregnancy or lactation; ④ history of heavy drinking recently; ⑤ severe central nervous system disease and severe mental illness.

Discharge criteria: ① New arrhythmias and ST changes were detected after entering the room; ② Accidents such as allergy and hypoglycemic coma occurred during operation waiting; ③ Other unexpected circumstances.

### Randomization and blinding

This was a double-blinded study. The participants, anesthesiologists were all blinded. Randomization was generated by the computer random-sequence. All participants meeting the inclusion criteria were randomly assigned to the Group L or Group C at a ratio of 1:1. After the patients were included, the groups were allocated according to the random numbers, and the nurse responsible for dispensing were informed. The nurse would prepare different experimental drugs according to different group, seal them and give them to the responsible anesthesiologist. The anesthesiologist would carry out the experiment according to the experimental steps and make records.

### Standard anesthesia procedures

According to our routine practice, after fasted for at least 8 h and arriving in the endoscopy room, the following parameters were continuously monitored, including: non-invasive blood pressure (NBP), heart rate (HR), electrocardiography (ECG), and pulse oxygen saturation (SpO_2_). Then, intravenous catheterization, left lateral position, and 6 L/min oxygen continuous inhalation via a facemask were supplied.

The experimental medicine was prepared by a specialized nurse: 2% lidocaine was diluted into 1% for Group L and saline was prepared for Group C. Then, within 10s, 0.15 ml/kg of the experimental drug was injected intravenously into the patients, that is, patients in Group L was given 1.5 mg/kg lidocaine (1% lidocaine), and group C was given the same amount of normal saline. 30s after experimental drug injection, an initial bolus of propofol was intravenously injected in 20s to induce sedation. According to the recommended dose of propofol, the initial induction dose of propofol for the first patient in each group was 1.5 mg/kg.

The depth of sedation was assessed according to the modified observer’s assessment of alertness/sedation (MOAA/S) score [8]: 5: response readily to name spoken; 4: lethargic response; 3: response after name called loudly; 2: response after mild to moderate shaking; and 1: response to trapezius squeeze. Until the patients’ MOAA/S score ≤ 1, the gastroscope will be inserted by a skilled endoscopist, who have completed more than 1000 procedures. A rescue bolus of 0.5 mg/kg propofol was injected if MOAA/S score ≥ 2 or the patients’ response was ‘movement’ during the procedure.

All adverse events during propofol sedation and gastroscopy were recorded and treated according clinical routine. Hypoxemia was defined as SpO_2_ below 90%, may treated with jaw-thrust maneuver, increasing the oxygen from 6 to 10 L/min, even assisted ventilation with a face-mask and withdrawal of endoscope. Bradycardia was defined if HR decreased to lower than 45 beats/min, and treated with 0.25–0.5 mg atropine intravenously as needed. Hypotension was defined if MAP fell below 65 mmHg, and 5–10 mg ephedrine was intravenously injected immediately if necessary.

### Outcome assessment

The primary outcome was the ED_50_ of propofol. ED_50_ of propofol was determined by the modified Dixon’s up-and-down method (MDUDM) [18]: if the response of the first enrolled patient was ‘no-movement’, the induction dose of propofol would be increased by 0.1 mg/kg in the subsequent patient, otherwise decreased. And the ‘movement’ responses were defined if cough, swallow, occurred during the endoscope insertion, in addition, if the MOAA/S score was still ≥2 after the initial propofol 2 min. In the MDUDM method, stop enrolling patients until at least six movement/no-movement pairs occur.

The secondary outcomes were the induction time, the FPBT, and the AEs. AEs during propofol sedation and gastroscopy were recorded and treated according to clinical routine.

### Statistical analysis

The ED_50_ of propofol was calculated by calculating the mean of midpoints of all crossover points acquired by the MDUDM [18]. And probit regression analysis was applied to obtain the doses of propofol where 50% (ED_50_) and 95% (ED_95_) of endoscope insertion attempts were successful.

SPSS 26.0 (IBM Inc., Armonk, NY, USA) was applied to analyze the results, *P* < 0.05 was considered statistically significant. ① Patients’ demographic data were recorded and presented as mean ± standard deviation or absolute numbers, and compared using the independent-sample t test or Chi-square test; ②The induction time, FPBT, MAP and HR in the two groups were expressed as mean ± standard deviation, analysed with the independent-sample t test; ③ The occurrence of AEs was expressed as number (n) of patients during sedation and endoscope insertion, and analysed with Chi-square test.

## Results

**Fig. 1:**
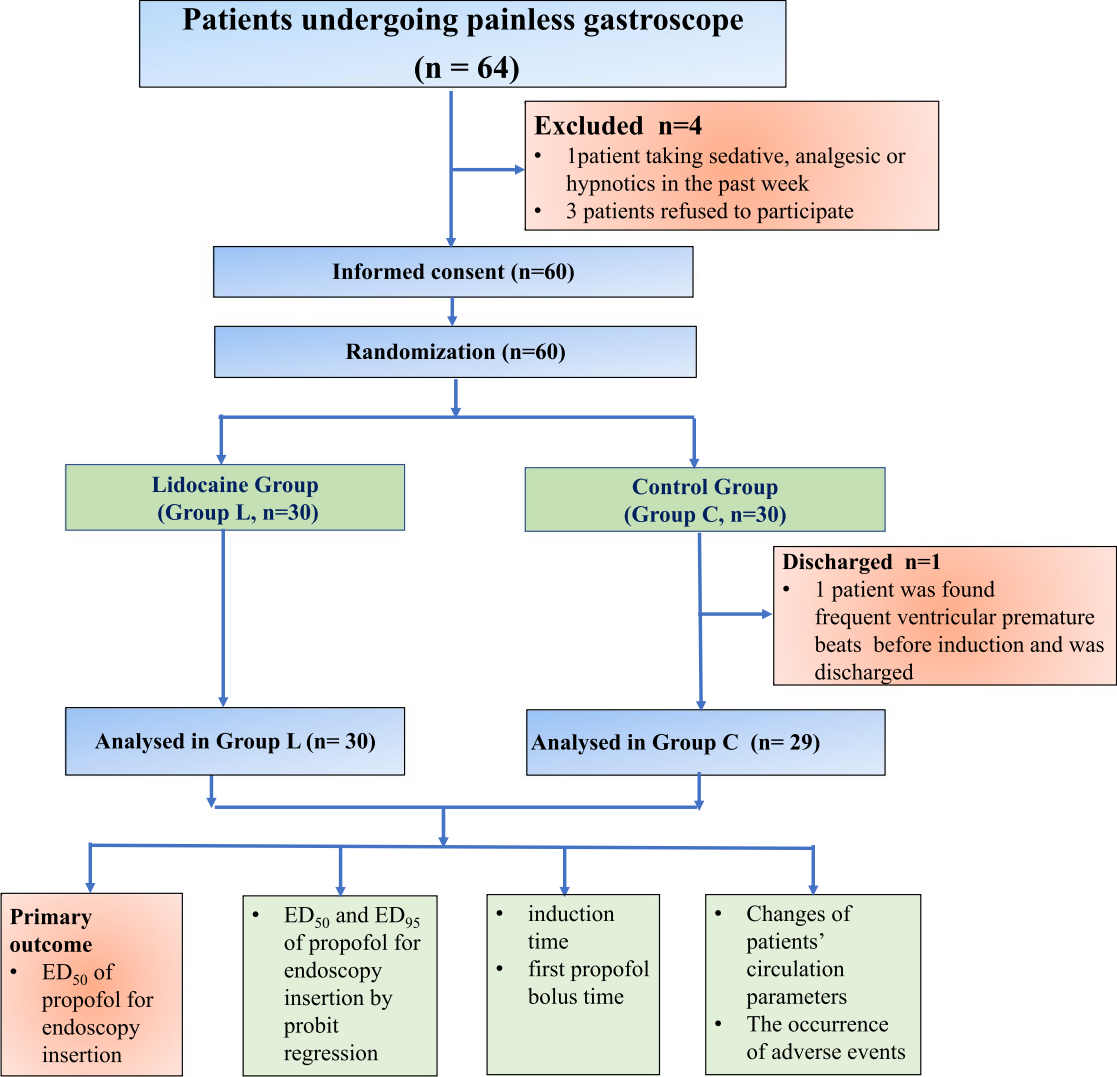
Flow chart of allocation of patients

This study was performed between May 2022 and June 2022. A total of 59 patients were enrolled and completed the study, 30 patients in Group L, 29 patients in Group C, as the flow chart shown in Fig. 1. There was no significant difference in demographic information between the two groups (Table 1).

**Table 1:** Patients’ demographic data

Parameters	Overall	Group L (n = 30)	Group C (n = 29)	***P*** value
**Sex (male/female)**	24/35	12/18	12/17	0.914
**Age (years)**	40 ± 7	40 ± 6	41 ± 7	0.704
**Height (cm)**	166 ± 9	167 ± 8	166 ± 10	0.510
**Weight (kg)**	67 ± 13	68 ± 13	66 ± 13	0.417
**BMI (kg/m**^**2**^**)**	24 ± 3	24 ± 3	24 ± 3	0.546
**ASA status (I/II)**	40/19	22/8	18/11	0.355
**Cigarette smoking history**				
No	52	25	27	0.136
≤ 10 cigarette rolls	3	1	2	
> 10 cigarette rolls	4	4	0	
**Alcohol intake history**				
No	49	25	24	1.000
≤ 76 g	5	2	3	
> 76 g	5	3	2	
**Comorbidities**				
No	54	28	26	0.704
Hypertension	3	1	2	
Hypothyroidism	1	1	0	
Diabetes	1	0	1	

Data are presented as the mean ± SD or absolute numbers

**Fig. 2:**
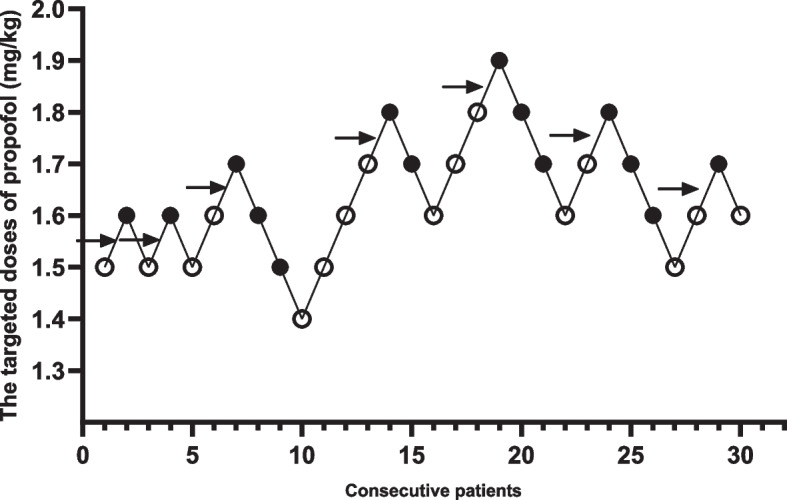
Response of patients with the modifiled Dixon’s up-and-down method in Group L. Responses of 30 consecutive patients in Group L to endoscope insertion and their initial doses of propofol are shown. Arrow indicates the midpoint dose of all independent pairs of patients who manifested crossover from ‘movement’ (○) to ‘no movement’ (●) responses

**Fig. 3:**
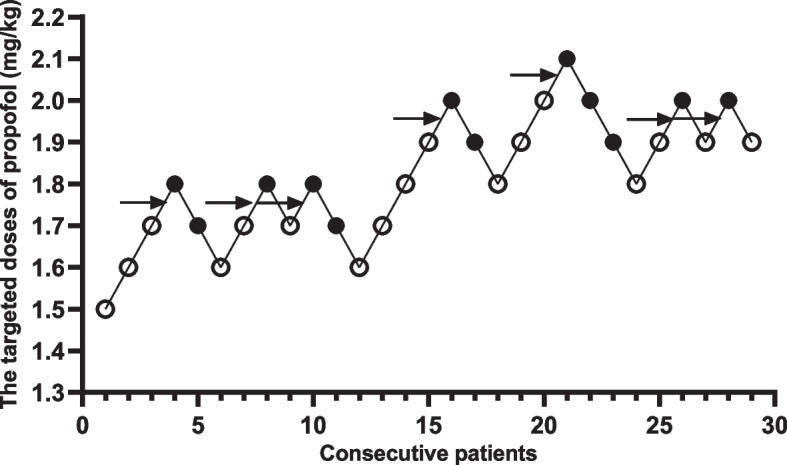
Response of patients with the modifiled Dixon’s up-and-down method in Group C. Responses of 29 consecutive patients in Group C to endoscope insertion and their initial doses of propofol are shown. Arrow indicates the midpoint dose of all independent pairs of patients who manifested crossover from ‘movement’ (○) to ‘no movement’ (●) responses

**Fig. 4:**
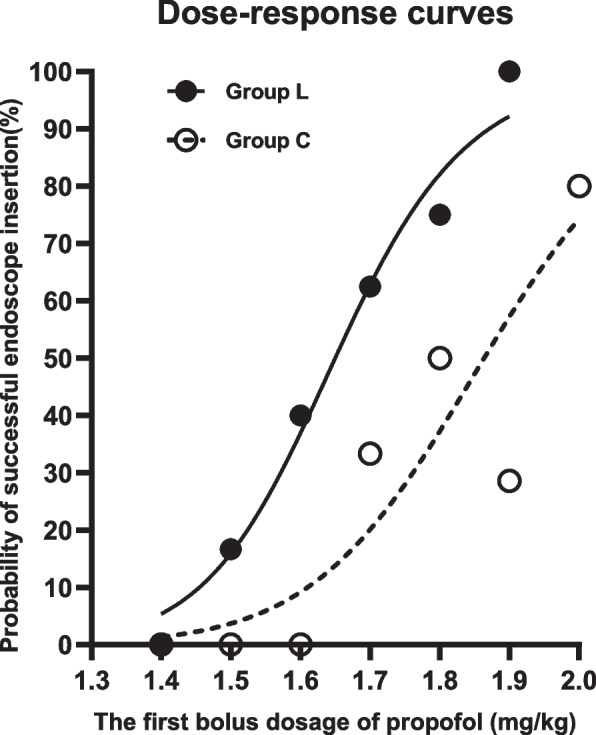
Dose-response curves of propofol for successful endoscope insertion

The ED_50_ of propofol for successful endoscope insertion in the Group L was 1.68 ± 0.11 mg/kg, which was significantly lower than that in Group C, 1.88 ± 0.13 mg/kg (Figs. 2 and 3). By the probit regression analysis, ED_50_ and ED_95_ of propofol for successful endoscope insertion in Group L were 1.65 mg/kg (95% confidence interval, 1.54–1.81 mg/kg) and 1.92 mg/kg (95% confidence interval, 1.78–3.10 mg/kg), respectively. By the same analysis, ED_50_ and ED_95_ of propofol for successful endoscope insertion in Group C were 1.86 mg/kg (95% confidence interval, 1.74–2.10 mg/kg) and 2.20 mg/kg (95% confidence interval, 2.02–3.46 mg/kg), respectively. The dose-response curves of propofol for successful endoscope insertion in both groups drew from the probit regression analysis were shown in Fig. 4.

There was no statistical difference in induction time and the first propofol bolus time between the two groups (Table 2).

**Table 2:** The Induction time and the first propofol bolus time (FPBT)

Items	Group L	Group C	***P*** value
Induction time (s)	87.9 ± 15.0	94.5 ± 16.4	0.115
FPBT (s)	182 ± 56 (n = 14)	169.2 ± 110 (n = 12)	0.655

Values are expressed as mean ± SD

In comparison with the time before induction, the MAP was obviously lower after sedation in both groups, and the MAP of lidocaine group was significantly lower than mono-propofol group after sedation. Besides, two patients’ MAP after sedation were below 65 mmHg in Group C, none in Group L. There was no significant difference in HR between the two groups before induction, and there was no significant change in HR after sedation compared with the time before induction (Table 3).

**Table 3:** Changes in patient vital signs

Items	Time points	Group L	Group C	***P*** value
MAP (mmHg)	Before induction	93 ± 11	92 ± 9	0.751
	After sedation	85 ± 11	76 ± 10^*^	0.003
HR (bpm)	Before induction	87 ± 13	82 ± 13	0.152
	After sedation	86 ± 11	80 ± 14	0.078

Date is presented as the mean ± SD

Compared with before induction, *P < 0.05

During propofol sedation, if SpO_2_ ≤ 90% was used as standard to compare, there was no difference in the two groups. But, in comparison with the control group, the number of patient’s SpO_2_ ≤ 95% was significantly lower in lidocaine group. Only two patients in the Group L developed SpO_2_ ≤ 95%, while nine in the Group C. However, there was no statistical difference about the number of MAP ≤65 mmHg between the two groups, four patients’ MAP developed below 65 mmHg in the control group, while only one in the lidocaine group. There was no statistical difference on body movement between the two groups. In addition, no related side effects of lidocaine toxicity reaction occurred in the Group L (Table 4).

**Table 4:** The occurrence of AEs during sedation and endoscope insertion

	Group L (n = 30)	Group C (n = 29)	***P*** value
**Propofol sedation**			
SpO_2_ > 95%	28	20	0.039
SpO_2_ ≤ 95%	2	9	
SpO_2_ > 90%	29	28	1.000
SpO_2_ ≤ 90%	1	1	
**Endoscope insertion**			
No-movement	14	12	0.795
Movement^a^	16	17	
**Lidocaine toxicity reaction**	0	–	

Data are presented as absolute numbers

^a^Movement means cough, swallow or body movement

## Discussion

The initial dose of propofol in our study was set as 1.5 mg/kg, and the reasons are as follows: on the one hand, according to Liu Fuk’s previous experience [18], their initial dose of mono-propofol for the first patient was set as 1.6 mg/kg; on the other hand, the minimum recommended dose of propofol induction is 1.5 mg/kg; at last, we hypothesized that lidocaine could reduce propofol consumption, so the initial dose of propofol in our study was set as 1.5 mg/kg.

The main outcome of this study, the ED_50_ of propofol combined with intravenous 1.5 mg/kg lidocaine, was 1.68 ± 0.11 mg/kg, which could decrease the ED_50_ of propofol mono-inducing dose approximately 0.24 mg/kg, or 12.8%, for successful endoscope insertion in adult patients. Those were who did not take local anesthetic in the past 24 hours, or analgesics and hypnotics in the past 7 days. But, the assumption of ED_50_ is mean that another 50% of patients will still failure for endoscope insertion. So, we utilized the probit regression analysis to determine the ED_95_ of propofol combined with intravenous lidocaine, 1.92 mg/kg, which was lower than that in propofol mono-inducing population, 2.20 mg/kg.

The second outcome of this study was the induction time and the first propofol bolus time, and there was no significant difference between the two groups. It can be said that adding lidocaine and reducing propofol consumption could not extend the induction time and shorten the first propofol bolus time under the same depth of sedation.

Recently, a number of studies have been published and demonstrated that perioperative lidocaine infusion is effective in reduction of postoperative pain, nausea, ileus duration, opioid requirement, ect [19–22]. How these benefits occur at the relatively low blood concentrations and how they persist for hours, it is a challenge to explain. This mechanism may not be mainly sodium channel blockade, it seems possible to interfere with other molecular targets, especially those involved in inflammatory signaling [23]. And neuronal effects may also play a role [24]. The toxicity of perioperative infusion of lidocaine is extremely rare, but tinnitus, perioral numbness and arrhythmia may occur [24]. For patients at increased risk of lidocaine toxicity, such as those with abnormal liver or renal functions or those who cannot ask for symptoms of lidocaine toxicity, monitoring of plasma lidocaine levels may be considered [25]. Fortunately, in this study, lidocaine was injected intravenously only once, and the dose was 1.5 mg/kg, the same as that of other similar studies [26, 27], which would not cause toxic reactions to patients.

However, Haoran Liu [17] et al. has found that the ED_50_ of propofol with a single intravenous bolus of 1.5 mg/kg lidocaine was 1.69 mg/kg [95%CI (1.62–1.78) mg/kg] in adult patients for gastroscopy, while sufentanil also worked. As propofol combined with sufentanil not only results in better analgesia, less cough, and lower propofol dosage, but also more severe respiratory depression sometimes [8–10]. Besides, the population in this study, whether exclude who used local anesthetic or had history of heavy drinking recently, or analgesics and hypnotics in the past 7 days was not clear. The ED_50_ of propofol with a single intravenous lidocaine has not been reported in the literature. So, we designed this trial. And, our main research results also confirmed this.

Liu Fuk [18] et al. had reported that the ED_50_ and ED_95_ of propofol for successful endoscope insertion were 1.90 mg/kg (95% CI, 1.78–2.10 mg/kg) and 2.15 mg/kg (95% CI, 2.01–3.56 mg/kg) respectively by the probit regression analysis. As expected, our findings were consistent with the above findings, 1.86 mg/kg (95% confidence interval, 1.74–2.10 mg/kg) and 2.20 mg/kg (95% confidence interval, 2.02–3.46 mg/kg) respectively in our study.

The recorded HR did not show any statistical difference between the two groups at the same time or after sedation, the result means that addition of lidocaine show no significant cardiac conduction system inhibition. While, the results of MAP between the two groups showed addition of lidocaine could reduce the haemodynamic fluctuations by reducing propofol requirement.

Although, there were no statistical difference about the occurrence of movement and the number of patient’s SpO_2_ ≤ 90% in the both groups, the number of patient’s SpO2 ≤ 95% was significantly lower in lidocaine group. It seems that intravenous lidocaine could relieve the degree of respiratory depression by reducing the propofol consumption, which adhere to our original hypothesis. Although the number of cases of this study was small, we did not obtain the effect of lidocaine on the incidence of desaturation events. However, this result gave us confidence and laid a foundation for the next step to observe the advantages of combined with lidocaine in painless gastrointestinal endoscopy in large sample study in future.

This study had some limitations. Firstly, there was no objective indicators of sedation level was measured during the procedure, such as BIS or EEG monitoring. As we applied MOAA/S score, the subjective observation techniques, as our sedation indicator, there might be slightly differences in the judgment of sedation depth; Secondly, this study only recruited relatively healthy patients (ASA I or II) with normal liver and kidney function. We did not extend to older or more severely ill patients (ASA III or IV), who were more likely to suffer respiratory and cardiovascular depression when exposed to propofol and more sensitive to IV lidocaine.

## Conclusions

In conclusion, the ED_50_ of propofol combined with intravenous lidocaine for successful endoscope insertion in adult patients, was significantly reduced compared with the normal saline. Adding lidocaine and reducing propofol consumption did not extend the induction time and not shorten the length of the FPBT under the same depth of sedation. At the same time, intravenous lidocaine may relieve the degree of respiratory depression by reducing the propofol consumption.

## Data Availability

The data sets used and/or analyzed during the present study are available from the corresponding author on reasonable request. The email of corresponding author is anlixin8120@163.com.

## Abbreviations

ERCP: Endoscopic retrograde cholangiopancreatography
ED_50_: 50% effective dose
GI: Gastrointestinal
BP: non-invasive blood pressure
HR: heart rate
SpO_2_: pulse oximetry
BMI: Body Mass Index
AEs: adverse events
